# Supplementary material for: Brain network dynamics during working memory are modulated by dopamine and diminished in schizophrenia
Source: Nat Commun. 2021 Jun 9;12:3478. doi: 10.1038/s41467-021-23694-9 (PMC8190281; doi:10.1038/s41467-021-23694-9)
Supplement: Supplementary file 1 — Supplementary Information [file 41467_2021_23694_MOESM1_ESM.pdf]

SUPPLEMENTARY INFORMATION

1. Supplementary Methods
1.1. Participants and study design
1.2. Data acquisition
1.2.1. N-back paradigm
1.3. Atlas construction
1.4. Network Control Theory
1.4.1. Model assumptions
The framework of network control theory that we have employed here is based on linear system model that relies on several assumptions:
1.4.2. Continuous versus discrete dynamical models
1.4.3. Stabilization of the dynamical system
1.4.4. Time horizon
1.4.5. On the relationship between BOLD signal and control theory measures
1.5. On the use of control theory as a statistical framework
1.6. Gene based polygenic co-expression indices
1.6.1. Polygenic co-expression index calculation
2. Supplementary Results
2.1. Null models of structural brain networks
2.2. Null models of spatial activity patterns
2.3. Robustness to choice of parcellation scheme
2.4. Robustness to choice of edge definition
2.5. Impact of medication and duration of illness on control properties
2.6. Pharmacological validation using Risperidone
2.7. Null results for gene score and imaging associations
2.8. Relation to previous gene score and imaging associations
2.9. Comparison to more conventional SPM analyses
2.10. Suboptimal trajectories
3. Supplementary figures
3.1. Supplementary Figure 1: N-back task design
3.2. Supplementary Figure 2: Control Energy, Stability and Average Brain Activity for Placebo and Amisulpride
3.3. Supplementary Figure 3: Control Energy, Stability and Average Brain Activity for Individuals with schizophrenia and Healthy Controls
3.4. Supplementary Figure 4: Control Impact for different visualization thresholds
4. Supplementary tables
4.1. Supplementary Table 1: Statistical details for the main findings and replication analyses
4.2. Supplementary Table 2: Graph metrics for structural connectomes
Supplementary Table 3: List of brain regions included in the extended Glasser parcellation
5. Supplementary references

1. Supplementary Methods

1.1. Participants and study design

All participants provided written informed consent for protocols approved by the Institutional Review Board of the medical faculty in Mannheim. For the first study including healthy controls and patients with schizophrenia, we included a total of 202 subjects (178 healthy controls, 24 individuals with schizophrenia, see Supplementary Table 1). General exclusion criteria in controls included the presence of a lifetime history of psychiatric, neurological, or significant general medical illness, pregnancy, a history of head trauma, and current alcohol or drug abuse. The patients were recruited from the Department of Psychiatry and Psychotherapy at the Central Institute of Mental Health in Mannheim and via local advertisements. A trained psychiatrist or psychologist verified the diagnosis of schizophrenia based on ICD-10 criteria.

Neuropsychological characterization of healthy controls included the trail-making-test B (TMT-B) (1) and the German multiple-choice vocabulary intelligence test (MWT-B) (2) as a measure of premorbid intelligence. Clinical characterization included the assessment of current symptom severity using the Positive and Negative Symptom Scale (PANSS) (3), Beck's Depression Inventory (4), measures of global functioning in daily life (global assessment of function (GAF)) and current antipsychotic medication dosage (converted into chlorpromazine dose equivalents (CPZE)).

For the second, pharmacological intervention study, 17 healthy individuals completed a subject- and observer-blind, placebo-controlled, randomized three-period cross-over study (see Table 2). Exclusion criteria included a regular consumption of drugs or history of drug or alcohol abuse; systolic blood pressure (SBP) greater than 140 or less than 90 mm Hg, and diastolic blood pressure (DBP) greater than 90 or less than 50 mm Hg; notable resting bradycardia (heart rate (HR) <40 bpm) or tachycardia (HR >90 bpm); use of any medication or herbal remedies taken within 14 days prior to randomization into the study or 5 times the elimination half-life of the medication, clinically significant abnormalities in laboratory test results (including hepatic and renal panels, complete blood count, chemistry panel and urinalysis): a history or presence of clinically significant ECG abnormalities (e.g. PQ/PR interval >210 ms, QTcF >450 ms) or cardiovascular disease (e.g. cardiac insufficiency, coronary artery disease, cardiomyopathy, hypokalemia, congestive heart failure, family history of congenital long QT syndrome, family history of sudden death); any personal or familial history of seizures, epilepsy or other convulsive condition, previous significant head trauma, or other factors predisposing to seizures; disorders of the central nervous system, cerebrovascular events, Parkinson's disease, migraine, depression, bipolar disorder, anxiety, any other psychiatric disorders or behavioral disturbances; regular smoking (>5 cigarettes, >3 pipe-fulls, >3 cigars per day); habitual caffeine consumption of more than 400 mg/d (approximately 4 cups of coffee or equivalent); a history or evidence of any clinically significant endocrinological, hepatic, renal, autoimmune, pulmonary, gastrointestinal, urogenital, oncological, hematological or any other disease; or a body mass index (BMI) of over 30 or below 22.

Participants were invited for a fixed interval of 7 days with each scanning session taking place at approximately the same time of day. On each of three scanning visits, individuals either received a single oral dose of 400 mg Amisulpride, 3 mg Risperidone or Placebo. MRI scanning took place 2 hours after drug administration, with the N-back paradigm commencing approximately 10 minutes after the start of the scan. One subject was excluded from the analysis due to an excessive body-mass index (BMI > 30).

1.2. Data acquisition

1.2.1. N-back paradigm

The visual N-back paradigm is a well-established and reliable working memory task consisting of a high memory load (2-back) and an attention control condition requiring motor response (0-back) (5-7). Specifically, a diamond-shaped stimulus containing a number from 1 to 4 was presented every 2 seconds (see Supplementary Figure 1). In the 0-back condition, subjects were required to press the button on the response box corresponding to the number currently displayed on the presentation screen. In the 2-back condition, subjects were required to press the button on the response box corresponding to the number presented two stimuli before the number currently displayed on the presentation screen. Stimuli were presented in alternating blocks of either 0-back or 2-back conditions. In each condition block, 14 stimuli were presented. Each condition block was repeated 4 times. Task performance was measured by accuracy (defined as the percent of correct answers) and reaction time (defined as the time span between stimulus onset and button press) for each condition separately.

1.3. Atlas construction

To combine structural and functional brain imaging data, we first constructed a brain atlas that equally well respects functional and anatomical features. We transformed a recently published multimodal atlas (8) into a volumetric format by projecting its FreeSurfer pial cortex coordinates into standard MNI space. A grey matter prior probability map (thresholded at 0.3) provided in SPM was used to define relevant voxels. Voxels were labeled by choosing the closest label with maximum distance of 4 mm. Since the published multimodal atlas does not cover all subcortical regions of interest (e.g. amygdala, thalamus), we complemented it with subcortical structures from the Harvard-Oxford atlas as implemented in FSL (9). Combining the two atlases resulted in 374 regions that covered cortical and subcortical structures. A full list of regions included in the combined atlas can be found in Supplementary Table 3.

1.4. Network Control Theory

1.4.1. Model assumptions

The framework of network control theory that we have employed here is based on linear system model that relies on several assumptions:

- Linearity: Linearity implies that the system evolves linearly over time. This is probably not an accurate description of most brain dynamics, but non-linear dynamics can be locally approximated by linear dynamics for macroscale brain circuits (10, 11).
- Time invariance: Time invariance means that a systems response does not depend on the time point because both the structural network A and the control set B are constant over time. As we consider brain dynamics over the time scale of minutes, both assumptions hold true.
- Freedom from noise: Neural systems at all time and spatial scale are not noise free. Nevertheless, it seems reasonable to consider that the salient features of our model do no depend on noise. This is aided by our definition of brain states as meta states that are defined as statistical patterns of brain activation over repeated measurements.

1.4.2. Continuous versus discrete dynamical models

Linear dynamical system can be studies using different time-system, either continuous or discrete. A discrete-time system assumes that the system evolves in discrete time steps whereas a continuous-time system models continuously changing dynamics. The choice depends on which time assumptions best reflects the neural dynamics at study. Our choice was motivated by a) our definition of brain states as non-discrete entities of dimensional brain activity summarized over extended brain region and b) the computational traceability. As we considered brain regions containing millions of neurons as network nodes, we assumed that each brain region`s state change is more heterogenous and therefore better represented as a continuous system. For reasons of computational feasibility, we used a discrete-system approach for the computation of the suboptimal trajectories.

1.4.3. Stabilization of the dynamical system

If not stabilized, a dynamical system could potentially increase infinitely over time. Such extreme brain states would be neurobiologically implausible due to the finite energy resources of the brain. We therefore chose to normalize the system by decreasing the average weight of the connectome such that it goes to zero over time:

(1) $\mathbf{A}_{\mathrm{norm}} = \frac{\mathbf{A}}{|\lambda(\mathbf{A})_{\max}| + c} - \mathbf{I}$

Here, $\mathbf{I}$ denotes the identity matrix, $\mathbf{A}$ denotes the structural connectivity matrix and $|\lambda(\mathbf{A})\mathrm{max}|$ denotes the largest eigenvalue of the system. To normalize the system, we must specify the parameter *c*, which determines the rate of stabilization of the system. We choose *c* = 1, meaning that all modes decay and the system goes to zero over time. Within the range of brain states that converge to zero over time, we cannot make statements regarding whether any of these intermediate brain states are biologically plausible or are realized in human brains. Further work integrating experiment and theory is needed to more clearly define types of implausible states, and their respective mechanisms (e.g., metabolic, electrical, informational, or other physical constraints). A more in-depth and mathematical introduction and discussion can be found in Refs. (12-14).

1.4.4. Time horizon

The time horizon T specifies the time over which the control input is applied and the system can be pushed from one state to the other. It determines how quickly the system is required to converge and therefore small values might give the system insufficient time to reach the target state, making it hard to control. In theory, T is dimensionless if not coupled to external time domains. As we do not intend to model an evolving process in real time, we chose $T = 1$ to use a normalized time, in line with previous works (15), which allows the system to have adequate time to be controlled (12). For a systematic investigation of the influence of T on control processes in the context of brain networks, we refer the reader to (12), as well as to more general introductions of the control processes in linear dynamics (14, 16).

1.4.5. On the relationship between BOLD signal and control theory measures

In the control theory framework, the control input ($\mathbf{u}$) of a node and the state of that same node ($\mathbf{x}$) are highly interrelated. For example, if we consider a simplified system consisting of only one node, then the control energy E necessary to change the state of that node from an initial state ($\mathbf{x}_0$) to a target state ($\mathbf{x}_T$) is basically a function of the squared difference in that node's state

(2) $[\mathrm{E} \sim (\mathbf{x}_0 - \mathbf{x}_T)^2]$.

As our definition of brain states is based on β estimates that depend on BOLD activity, in such a simplified system control energy would not give any additional information other than the usual contrast images β_{2back}-β_{0back}. However, if we consider a more complex system with more than one node, and where all nodes are connected via either direct or indirect links as summarized in the connectivity matrix $\mathbf{A}$, then the control energy of a single node is not a simple function of the squared differences in its state but additionally accounts for the influence of other connected neighbors.

1.5. On the use of control theory as a statistical framework

In our analysis, we apply control theory as a statistical and theoretical tool to answer questions based on the theoretical "dual-state" framework regarding neurobiological properties of brain function. Translating

and transferring across these three levels (control theory as a statistical tool, dual-state theory as a non-linear theoretical framework, brain imaging data defining meta-level brain states) is challenging and requires (reasonable) simplifications. The hypotheses that we aim to test are based on the dual-state theory framework, which also uses the terminology of brain states and energy. In this framework, states and transitions are based on non-linear dynamics, corresponding to attractor basins, which translate to stable reoccurring activation patterns in neuronal ensembles (17-19). Abstracting these concepts to large-scale dynamics of brain macro-circuits provides the underlying basis for the idea that we aim to investigate here: relatively stable "meta"-level brain activation patterns as identified by neuroimaging (including all the caveats of the assumption of stationarity of brain activations measured by functional magnetic resonance imaging) populate a state-space for which we aim to identify the brain regions that are responsible for maintaining and shifting those activation patterns. To answer these cognitive neuroscience questions, we use *network control theory* as a toolkit that makes these questions computationally tractable in a linear dynamical system framework enabling us to quantify the associated "energy cost" of transitions on a brain region level. This effort requires certain (reasonable) assumptions, in particular to assume an equivalence between states defined by neuroimaging and states defined in the control theory framework, as well linear and continuous transitions between those states. Future work integrating biophysical models of task-induced brain activity in combination with network control theory and tailored imaging paradigms is critically needed to provide further evidence for the assumed relationships (and distinctions) between actual data, network control tools, and the theoretical framework.

1.6. Gene based polygenic co-expression indices

1.6.1. Polygenic co-expression index calculation

Previous publications have shown that gene sets defined using co-expression networks and selected for their association with the genes DRD1 and DRD2 provided replicable predictions of n-back-related brain activity and behavioral indices (20-23). Weighted Gene Co-expression Network Analysis [WGCNA (24)] applied on the Braincloud dataset (N=199) of post-mortem DLPFC gene expression (25) identified 67 non-overlapping sets of genes based on their expression pattern. The co-expression gene sets including DRD1 and DRD2 were summarized into Polygenic Co-expression Indices (PCIs) based on SNPs that predicted co-expression of these genes (called co-expression quantitative trait loci, or co-eQTLs).
PCIs are a proxy for the assessment of the genetic component of gene transcription co-regulation and are computed as a weighted average of the effect of all genotypes of an individual among those selected in the data mining study as co-eQTLs. The effect of individual SNPs is computed as the difference between the gene co-expression distribution of minor allele carriers (heterozygotes and homozygotes) and that of major allele homozygotes, using common tools from signal detection theory (26). Genotype weights, therefore, represent the deviation in gene co-expression from a reference distribution and are not constrained by allele dose. For each genotype of each SNP we computed an index, called A', proportional to the expression of the gene of interest (DRD1 or DRD2) within its co-expression module. The A' index is less dependent than d' on the assumption of a normal distribution of gene expression in each genotypic

population (20). Both PCI-based predictions were significantly replicated in an independent post-mortem dataset, while controlling for ethnicity. The translational effect of these two scores on brain activity during n-back has been assessed and replicated across multiple samples, which combined amount to approximately 600 participants (23, 27, 28).

It is important to note that these dopamine-related genetic effects are large in magnitude compared to those estimated by polygenic risk score approaches that focus on epidemiological data, rather than on molecular processes. The DRD2-PCI we developed (23) yielded an effect size $f = 0.30$ in our n-back discovery sample (required sample size to obtain 80% power with $\alpha = 0.05$ and covariates as in the current work: $N = 71$). Results were replicated in an independent fMRI dataset collected at a different institution with $f = 0.20$ (required sample size computed as above: $N = 156$). Our follow-up work on the DRD2-PCI (21) considered two datasets of 50 individuals each and yielded a minimum effect size $f = 0.28$ (in the replication sample; required sample size computed as above: $N = 81$). The DRD1-PCI was also tested in two independent samples (20), yielding a minimum effect size $f = 0.37$ (in the replication sample; required sample size computed as above: $N = 46$). Taken together, these published results show that the effects of these polygenic indices on n-back activity in the prefrontal cortex are relatively large, with sizes ranging between 0.20 and 0.37 and with required samples ranging from 46 to 156 individuals. Importantly, the DRD2-PCI was also tested in a small sample of 29 patients with SCZ and yielded results consistent with the effects discovered in healthy controls (23). Although the required sample sizes were computed based on the top cluster, it should be borne in mind that the technique we used in this work employs the entire brain, and therefore (i) is not subject to correction for multiple comparisons, as reflected in the uncorrected alpha used for the power calculations and (ii) benefits from the greatest possible amount of information about brain states.

2. Supplementary Results

2.1. Null models of structural brain networks

To study the impact of structural brain networks on control properties, we repeated the computation of control energy using a randomized null model of the individuals' structural brain networks that preserves the degree distribution and ensure fully connected networks. Null models were created using the *randmio_und_connected* function, rewiring each edge 20 times, as implemented in the Brain Connectivity Toolbox (https://sites.google.com/site/bctnet/). For each subject, we created 100 null models and recomputed control energy. The average control energy over 100 null models was used for further analyses. In line with our expectation, control energy increased significantly for randomized networks (repeated measures ANOVA with null_model_vs_data and transition as within-subject factors: main effect of null_model_vs_data, $F(1,174) = 5.183$, $p = 0.024$). Further analysis revealed no interaction with patient status (repeated measures ANOVA with null_model_vs_data and transition as within-subject factors and

group as between-subject factor: group by null_vs_data interaction, F(1,99) = 0.289, p = 0.592). These analyses suggest that human brain structural networks are in some form optimized to control brain state transitions independent of diagnostic status.

2.2. Null models of spatial activity patterns

To study the impact of the spatial distribution of activity patterns on control properties, we repeated the computation of control energy and spatially randomized individuals' brain activation patterns. Randomization was done using the *randperm* function in matlab for the paired vectorized brain activation patterns (related to 0- and 2-back) followed by recomputation of control energy. This procedure was repeated 100 times and the averaged brain-wide control energy over all 100 iterations for each subjects was used in the subsequent analysis. In line with our expectation, control energy increased significantly for randomized networks in both groups (repeated measures ANOVA with model_vs_data and transition as within-subject factors, HC: main effect of model_vs_data, F(1,174) = 6.995, p = 0.009). Further analysis revealed no interaction with patient status (repeated measures ANOVA with null_model_vs_data and transition as within-subject factors and group as between-subject factor: group by null_vs_data interaction, F(1,99) = 3.904, p = 0.056). These analyses suggest that the spatial distribution of brain activity patterns is important for minimizing control effort, but individuals with schizophrenia have a differently, potentially less organized activity pattern than healthy controls.

2.3. Robustness to choice of parcellation scheme

To demonstrate the robustness of the results to our choice of parcellation scheme, we repeated our analysis using a recently published functionally defined atlas comprising a similar number of areas (29). Specifically, we used the "Gordon" template (29) consisting of 333 regions that are functionally derived from resting-state connectivity analyses. Data were reprocessed using the same pipeline as for the main analysis and all parameters were kept identical in the subsequent analysis. Notably, we replicated all main results (see Supplementary Table 1), indicating that our reported findings are robust to the choice of parcellation scheme.

2.4. Robustness to choice of edge definition

To demonstrate the robustness of our results to our selection of connectivity measure, we repeated our analysis using the number of streamlines normalized by the respective size of the regions to construct structural connectivity matrices (15). All parameters were kept identical in the subsequent analysis. All main results could be replicated (see Supplementary Table 4), indicating that our findings are robust to the choice of edge definition.

2.5. Impact of medication and duration of illness on control properties

In patients, the potential relationship between control energy and stability, antipsychotic drug dose (expressed in chlorpromazine equivalents (CPZE), n=20), and clinical parameters (illness duration, illness

severity as indexed by global functioning (GAF) and Positive and Negative Symptom Scale (PANSS)) were explored using Pearson correlation. Neither the control energy for the 0-back to 2-back transition nor the opposite transition or the stability of either state were significantly associated with CPZE (N = 20, 0- to 2-back: r = 0.078, p = 0.767; 2- to 0-back: r = 0.320, p = 0.210; 0- back stability: r = 0.150, p = 0.564; 2- back stability: r = 0.096, p = 0.713), with illness duration (N = 23, 0- to 2-back: r = 0.017, p = 0.937; 2- to 0-back: r = -0.226, p = 0.299; 0- back stability: r = 0.110, p = 0.644; 2- back stability: r = 0.281, p = 0.230), or with GAF (N = 24, 0- to 2-back: r = -0.086, p = 0.690; 2- to 0-back: r = -0.254, p = 0.230; 0- back stability: r = -0.135, p = 0.570; 2- back stability: r = 0.066, p = 0.793). Please note, that a lack of between-subject correlations in small samples can only provide weak proof of evidence for a null effect.

2.6. Pharmacological validation using Risperidone

To demonstrate the robustness of our pharmacological intervention of dopaminergic signaling, we additionally analyzed the data of the Risperidone condition in the same subjects. Risperidone also preferentially targets D2 receptors, but also affects D1, adrenergic, serotoninergic and histaminergic pathways. Using the same models and covariates as in the main analysis, we detected a trend-wise increase in control energy needed for both transitions (repeated measures ANOVA with drug and transition as within-person factors; main effect of drug: $F(1,10) = 3.490$, $p = 0.091$; drug-by-condition interaction: $F(1,10) = 0.238$, $p = 0.636$; activity difference, drug order, and sex as covariates of no interest), but no effect on stability ($F(1,8) = 0.105$, $p = 0.334$; mean brain activity, sex, and drug order as covariates of no interest). Although these results showed only trend-wise significance, likely due to the lower D2-specificity of Risperidone, the detected pattern was conserved across drugs, validating the proposed underlying concepts.

2.7. Null results for gene score and imaging associations

As mentioned in the main text, D1 receptor expression-related gene scores predicted stability of both states (0-back: b = 0.184, p = 0.034; 2-back: b = 0.242 p = 0.007), but not D2 receptor expression-related gene scores (0-back: b = 0.153, p = 0.109; 2-back: b = -0.01 p = 0.924). In turn, the control energy of both state transitions could be predicted by the D2 receptor expression-related score (0- to 2-back: b = -0.076, p = 0.037; and trending for 2- to 0-back: b = -0.134, p = 0.068), but not by the D1 receptor expression-related gene score (0- to 2-back: b = -0.037, p = 0.324; 2- to 0-back: b = -0.06, p = 0.418).

2.8. Relation to previous gene score and imaging associations

To demonstrate the added value of our analysis, we extracted the BOLD parameters from the voxels reported in Fazio et al. (30) and Selvaggi et al. (32) of a standard 2-back>0-back contrast image and included them a covariates in our main analysis.

D1 expression-related gene score predicted stability of both states (0-back: b = 0.184, p = 0.036; 2-back: b = 0.242, p = 0.008, age, sex, brain activity, first 5 genetic PCA components and brain activity at [-29 53 24] as peak voxel reported in Fazio et al. as covariates of non-interest).

D2 expression-related gene score predicted the control energy of both state transitions (0- to 2-back: b = -0.075, p = 0.042; 2- to 0-back: b = -0.139, p = 0.062, age, sex, brain stability, difference in brain activity, first 5 genetic PCA components and brain activity at [-29 53 24] as peak voxel reported in Fazio et al. as covariates of non-interest).

All our previous associations remained significant when controlling for the BOLD parameters, suggesting that our data explains different variance.

2.9. Comparison to more conventional SPM analyses

To demonstrate the added value of our control metrics, we performed the following two more conventional SPM analyses:

1) Association of control energy and stability with PCI scores for DRD1 and DRD2:
   We performed a conventional GLM analysis for Placebo versus Amisulpride, using a paired t-test of the 2-back>0back contrast in SPM12. We could not detect any significant clusters, either for the Placebo > Amisulpride nor the opposite contrast, even at a lenient threshold of P < 0.001 uncorrected. These results suggest that network control theory can detect biologically meaningful effects that cannot be detected by more conventional SPM analyses.
2) Stability and Control Energy in Schizophrenia:
   We performed a conventional SPM analysis for individuals with schizophrenia versus healthy control, using the 2-back > 0back contrast. At a lenient threshold of P < 0.001 uncorrected, we detected two main significant cluster of voxels in the common region-of-interests for the N-back task (comprising dorsolateral prefrontal cortex, hippocampus and parietal cortex). The first cluster for the HC > SZ contrast was located in the right hippocampus at 42 -37 -13 [number of significant voxels = 24, P_{FWE}=0.554, T=3.86], while the second cluster was found in the opposite contrast and was located in the right parietal cortex at 51 -43 17 [number of significant voxels = 214, P_{FWE}=0.171, T=5.35; see supplemental figure 4B]. To demonstrate that the control energy and stability values of our analysis are not associated with the more conventional activity measures, we extracted the peak voxel beta estimates of those coordinates and performed a partial correlation analyses, correcting for age, sex and group. For both peak voxels, we did not detect a significant association with our control indices (beta estimates at 42 -37 -13: 0-back stability: r_{par} = -0.005, p = 0.961; 2-back stability: r_{par} = 0.139, p = 0.167; 0- to 2-back control energy: r_{par} = -0.115, p = 0.253; 2- to 0-back control energy: r_{par} = 0.150, p = 0135. beta estimates at 42 -37 -13: 0-back stability: r_{par} = -0.079, p = 0.431; 2-back stability: r_{par} = -0.106, p = 0.293; 0- to 2-back control energy: r_{par} = -0.144, p = 0.151; 2- to 0-back control energy: r_{par} = -0.100, p = 0.318).

2.10. Suboptimal trajectories

As mentioned in the main text, the variability in suboptimal trajectories was greater in schizophrenia (rm-ANOVA: main effect of group: $F(1,98) = 4.789$, $p = 0.031$, controlling for age, sex, DTI tSNR, brain state activity difference). These results remained significant after additionally accounting for the stability of both states and for the control energy of both transitions (rm-ANOVA: main effect of group: $F(1,95) = 11.2$, $p = 0.001$).

3. Supplementary figures

3.1. Supplementary Figure 1: N-back task design

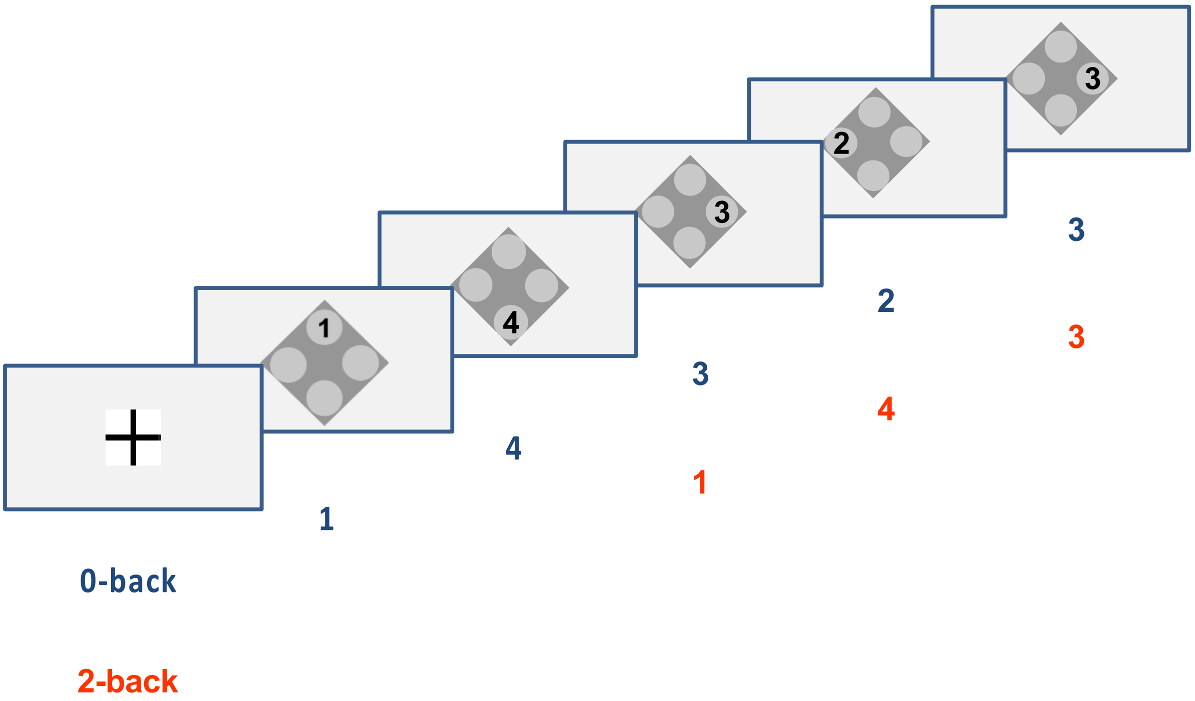

Design of the N-back task: Stimuli were presented in blocks of either 0-back or 2-back conditions. There was no additional control or resting condition. In the 0-back condition, subjects were instructed to press the button on the response box corresponding to the number currently displayed on the presentation screen. Here, the red numbers below the screen images indicate correct responses. In the 2-back condition, subjects were instructed to press the button on the response box corresponding to the number presented two stimuli before the number currently displayed on the presentation screen. Here, the red numbers below the screen images indicate the correct responses. Each condition block lasted 30 seconds and was repeated four times in an interleaved manner.

3.2. Supplementary Figure 2: Control Energy, Stability and Average Brain Activity for Placebo and Amisulpride

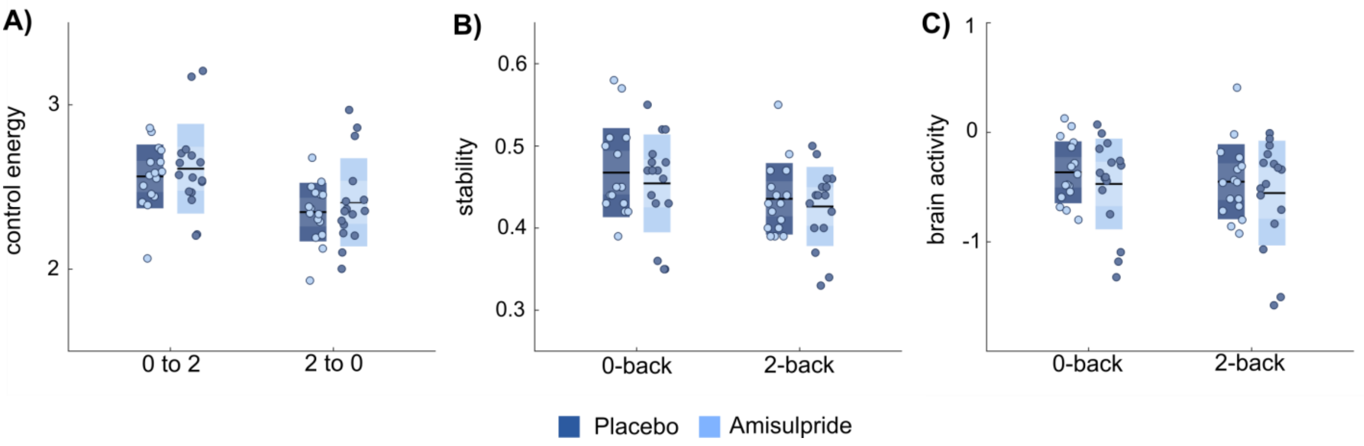

Supplementary Figure 2: Control Energy, Stability and Average Brain Activity for Placebo and Amisulpride

Plots depicting the actual data points n = 15 healthy controls for A) control energy, B) stability and C) average brain activity for the pharmacological study. All tests were two-sided and without adjustments for multiple comparisons. Black lines indicate mean, dark boxes indicate 1 standard deviation, light boxes indicate 1.96 SEM.

3.3. Supplementary Figure 3: Control Energy, Stability and Average Brain Activity for Individuals with Schizophrenia and Healthy Controls

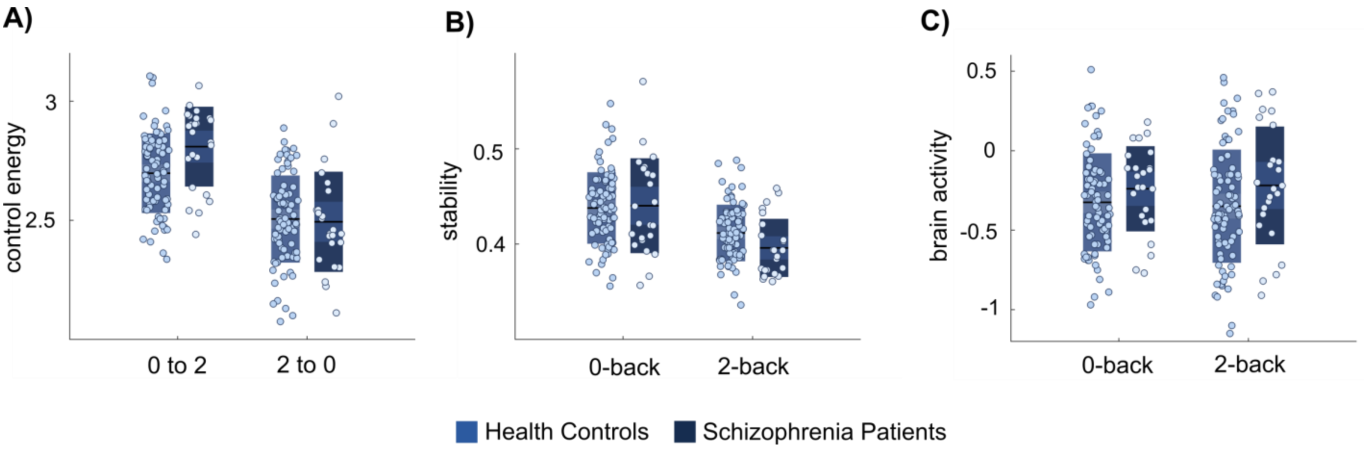

Supplementary Figure 3: Control Energy, Stability and Average Brain Activity for Individuals with Schizophrenia and Healthy Controls

Plots depicting the actual data points for A) control energy, B) stability and C) average brain activity for the schizophrenia patient study (healthy control: n = 80, individuals with schizophrenia: n = 24). All tests were two-sided and without adjustments for multiple comparisons. Black lines indicate mean, dark boxes indicate 1 standard deviation, light boxes indicate 1.96 SEM.

3.4. Supplementary Figure 4: Control Impact for different visualization thresholds

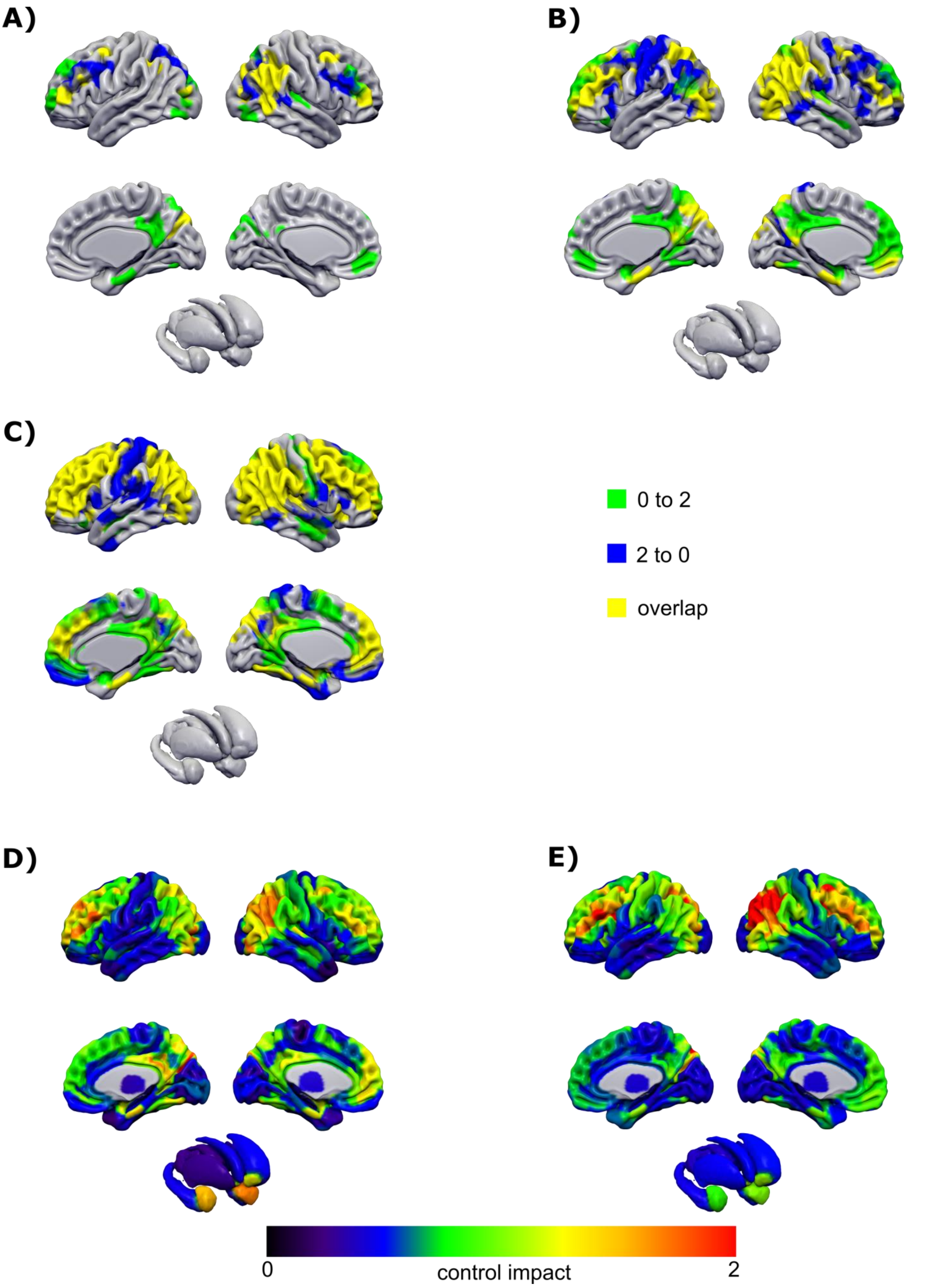

Supplementary Figure 4: Control Impact for different visualization thresholds

Unique and common sets of brain regions contributing most to the transition from 0-back to 2-back and the transition from 2-back to 0-back for different visualization thresholds derived from n = 178 healthy controls. For illustrative purposes, we projected the computed control impact of each brain region for the respective transitions on a 3D structural template, displaying A) the 10% highest for each transition B) the 30% highest for each transition and C) the 50% highest for each transition. D) and E) depict the raw control impact values for the transition from 0-back to 2-back or the transition from 2-back to 0-back, respectively.

4. Supplementary tables

4.1. Supplementary Table 1: Statistical details for the main findings and replication analyses

Result	Glasser						Gordon		
	FA			count			FA		
	df	F or t (stand beta)	p-val	df	F or t (stand beta)	p-val	df	F or t (stand beta)	p-val
General properties of control									
stability 0 back > 2 back	1,173	66.80	< 0.001	1,173	60.50	<0.001	1,173	56.846	<0.001
T02 > T20	1,174	27.98	<0.001	1,174	19.73	<0.001	1,174	21.706	<0.001
stability 2-back -> accuracy		-2.78 (0.274)	0.006		2.70 (0.252)	0.008		2.58 (-0.239)	0.011
stability 2-back -> RT		-1.94 (-0.192)	0.054		-1.94 (-0.182)	0.054		-1.95 (-0.181)	0.053
Differential relation to D1 and D2 expression									
D1 -> stability 0 back		2.18 (0.184)	0.034		2.212 (0.190)	0.031		3.10 (0.270)	0.003
D1 -> stability 2 back		2.78 (0.242)	0.007		2.978 (0.270)	0.004		3.39 (0.307)	0.001
D2 -> stability 0 back		1.629 (0.153)	0.109		1.963 (0.185)	0.055		0.146 (0.147)	0.149
D2 -> stability 2 back		-0.095 (-0.010)	0.924		0071 (0.008)	0.944		0.174 (0.019)	0.862
D2 -> T02		-2.14 (-0.076)	0.037		-2.33 (-0.09)	0.023		-2.07 (-0.07)	0.043
D2 -> T20		-1.87 (-0.134)	0.068		-1.83 (-0.134)	0.073		-2.16 (-0.152)	0.036
D1 -> T02		-0.996 (-0.037)	0.324		-1.066 (-0.045)	0.291		-0.832 (-0.029)	0.414
D1 -> T20		-0.817 (-0.06)	0.418		-0.650 (-0.049)	0.519		-0.376 (-0.028)	0.709
Drug effect on transition energy									
Pla vs. Ami	1,10	7.272	0.022	1,10	4.954	0.05	1,10	8.839	0.014
Pla vs. Ris	1,10	3.49	0.091	1,10	3.797	0.08	1,10	3.014	0.113
Drug effect on stability									
Pla vs. Ami	1,8	0.715	0.422	1,8	0.698	0.428	1,8	0.013	0.913
Pla vs. Ris	1,8	1.057	0.334	1,8	2.941	0.125	1,8	0.358	0.566
Schizophrenia									
stability 2-back: HC vs. SZ	1,98	6.436	0.013	1,98	6.552	0.012	1,98	4.951	0.028

stability 0-back: HC vs. SZ	1,98	0.041	0.840	1,98	0.105	0.746	1,98	0.327	0.569
T02: HC vs. SZ	1,98	5.238	0.024	1,98	6.414	0.013	1,98	5.070	0.027
T20: HC vs. SZ	1,98	0.620	0.433	1,98	0.534	0.467	1,98	0.275	0.601

Abbreviations: FA = structural edge weight defined as mean fraction anisotropy of a track connecting two regions; Count = structural edge weight defined as track count connecting two regions; Gordon = resting-state defined atlas with 333 regions; T02 = control energy for the transition 0 to 2-back; T20 = control energy for the transition 2 to 0-back; -> = predicts in a regression model; Q = modularity estimate; Pla = Placebo; Ami = Amisulpride; Ris = Risperidone; HC = healthy control; SZ = individuals with schizophrenia. All tests were two-sided and without adjustments for multiple comparisons

4.2. Supplementary Table 2: Graph metrics for structural connectomes

	Healthy controls (n = 178)	Matched controls (n = 80)	Individuals with schizophrenia (n = 24)	t value	P value
Network measures					
Density	0.053 ± 0.005	0.051 ± 0.006	0.051 ± 0.006	0.28	0.788
Average degree	18.51 ± 2.10	18.84 ± 2.25	18.72 ± 2.22	0.24	0.828
Global efficiency	0.15 ± 0.009	0.15 ± 0.11	0.15 ± 0.10	0.27	0.785
Average clustering coefficient	0.14 ± 0.009	0.14 ± 0.010	0.14 ± 0.009	0.78	0.437
Modularity (Q)	0.50 ± 0.03	0.50 ± 0.03	0.49 ± 0.03	0.988	0.326

All tests were two-sided and without adjustments for multiple comparisons

Supplementary Table 3: List of brain regions included in the extended Glasser parcellation

#	Brain Region Label
1	Glasser_L_V1
2	Glasser_L_MST
3	Glasser_L_V6
4	Glasser_L_V2
5	Glasser_L_V3
6	Glasser_L_V4
7	Glasser_L_V8
8	Glasser_L_4
9	Glasser_L_3b
10	Glasser_L_FEF
11	Glasser_L_PEF
12	Glasser_L_55b
13	Glasser_L_V3A
14	Glasser_L_RSC
15	Glasser_L_POS2
16	Glasser_L_V7
17	Glasser_L_IPS1
18	Glasser_L_FFC
19	Glasser_L_V3B
20	Glasser_L_LO1
21	Glasser_L_LO2
22	Glasser_L_PIT
23	Glasser_L_MT
24	Glasser_L_A1
25	Glasser_L_PSL
26	Glasser_L_SFL
27	Glasser_L_PCV
28	Glasser_L_STV
29	Glasser_L_7Pm
30	Glasser_L_7m
31	Glasser_L_POS1
32	Glasser_L_23d
33	Glasser_L_v23ab
34	Glasser_L_d23ab
35	Glasser_L_31pv
36	Glasser_L_5m
37	Glasser_L_5mv
38	Glasser_L_23c
39	Glasser_L_5L
40	Glasser_L_24dd
41	Glasser_L_24dv
42	Glasser_L_7AL
43	Glasser_L_SCEF
44	Glasser_L_6ma

45	Glasser_L_7Am
46	Glasser_L_7PL
47	Glasser_L_7PC
48	Glasser_L_LIPv
49	Glasser_L_VIP
50	Glasser_L_MIP
51	Glasser_L_1
52	Glasser_L_2
53	Glasser_L_3a
54	Glasser_L_6d
55	Glasser_L_6mp
56	Glasser_L_6v
57	Glasser_L_p24pr
58	Glasser_L_33pr
59	Glasser_L_a24pr
60	Glasser_L_p32pr
61	Glasser_L_a24
62	Glasser_L_d32
63	Glasser_L_8BM
64	Glasser_L_p32
65	Glasser_L_10r
66	Glasser_L_47m
67	Glasser_L_8Av
68	Glasser_L_8Ad
69	Glasser_L_9m
70	Glasser_L_8BL
71	Glasser_L_9p
72	Glasser_L_10d
73	Glasser_L_8C
74	Glasser_L_44
75	Glasser_L_45
76	Glasser_L_47l
77	Glasser_L_a47r
78	Glasser_L_6r
79	Glasser_L_IFJa
80	Glasser_L_IFJp
81	Glasser_L_IFSp
82	Glasser_L_IFSa
83	Glasser_L_p9-46v
84	Glasser_L_46
85	Glasser_L_a9-46v
86	Glasser_L_9-46d
87	Glasser_L_9a
88	Glasser_L_10v
89	Glasser_L_a10p
90	Glasser_L_10pp
91	Glasser_L_11l
92	Glasser_L_13l

93	Glasser_L_OFC
94	Glasser_L_47s
95	Glasser_L_LIPd
96	Glasser_L_6a
97	Glasser_L_i6-8
98	Glasser_L_s6-8
99	Glasser_L_43
100	Glasser_L_OP4
101	Glasser_L_OP1
102	Glasser_L_OP2-3
103	Glasser_L_52
104	Glasser_L_RI
105	Glasser_L_PFcm
106	Glasser_L_PoI2
107	Glasser_L_TA2
108	Glasser_L_FOP4
109	Glasser_L_MI
110	Glasser_L_Pir
111	Glasser_L_AVI
112	Glasser_L_AAIC
113	Glasser_L_FOP1
114	Glasser_L_FOP3
115	Glasser_L_FOP2
116	Glasser_L_PFt
117	Glasser_L_AIP
118	Glasser_L_EC
119	Glasser_L_PreS
120	Glasser_L_H
121	Glasser_L_ProS
122	Glasser_L_PeEc
123	Glasser_L_STGa
124	Glasser_L_PBelt
125	Glasser_L_A5
126	Glasser_L_PHA1
127	Glasser_L_PHA3
128	Glasser_L_STSda
129	Glasser_L_STSdp
130	Glasser_L_STSvp
131	Glasser_L_TGd
132	Glasser_L_TE1a
133	Glasser_L_TE1p
134	Glasser_L_TE2a
135	Glasser_L_TF
136	Glasser_L_TE2p
137	Glasser_L_PHT
138	Glasser_L_PH
139	Glasser_L_TPOJ1
140	Glasser_L_TPOJ2

141	Glasser_L_TPOJ3
142	Glasser_L_DVT
143	Glasser_L_PGp
144	Glasser_L_IP2
145	Glasser_L_IP1
146	Glasser_L_IP0
147	Glasser_L_PFop
148	Glasser_L_PF
149	Glasser_L_PFm
150	Glasser_L_PGi
151	Glasser_L_PGs
152	Glasser_L_V6A
153	Glasser_L_VMV1
154	Glasser_L_VMV3
155	Glasser_L_PHA2
156	Glasser_L_V4t
157	Glasser_L_FST
158	Glasser_L_V3CD
159	Glasser_L_LO3
160	Glasser_L_VMV2
161	Glasser_L_31pd
162	Glasser_L_31a
163	Glasser_L_VVC
164	Glasser_L_25
165	Glasser_L_s32
166	Glasser_L_pOFC
167	Glasser_L_PoI1
168	Glasser_L_Ig
169	Glasser_L_FOP5
170	Glasser_L_p10p
171	Glasser_L_p47r
172	Glasser_L_TGv
173	Glasser_L_MBelt
174	Glasser_L_LBelt
175	Glasser_L_A4
176	Glasser_L_STSva
177	Glasser_L_TE1m
178	Glasser_L_PI
179	Glasser_L_a32pr
180	Glasser_L_p24
181	Glasser_R_V1
182	Glasser_R_MST
183	Glasser_R_V6
184	Glasser_R_V2
185	Glasser_R_V3
186	Glasser_R_V4
187	Glasser_R_V8
188	Glasser_R_4

189	Glasser_R_3b
190	Glasser_R_FEF
191	Glasser_R_PEF
192	Glasser_R_55b
193	Glasser_R_V3A
194	Glasser_R_RSC
195	Glasser_R_POS2
196	Glasser_R_V7
197	Glasser_R_IPS1
198	Glasser_R_FFC
199	Glasser_R_V3B
200	Glasser_R_LO1
201	Glasser_R_LO2
202	Glasser_R_PIT
203	Glasser_R_MT
204	Glasser_R_A1
205	Glasser_R_PSL
206	Glasser_R_SFL
207	Glasser_R_PCV
208	Glasser_R_STV
209	Glasser_R_7Pm
210	Glasser_R_7m
211	Glasser_R_POS1
212	Glasser_R_23d
213	Glasser_R_v23ab
214	Glasser_R_d23ab
215	Glasser_R_31pv
216	Glasser_R_5m
217	Glasser_R_5mv
218	Glasser_R_23c
219	Glasser_R_5L
220	Glasser_R_24dd
221	Glasser_R_24dv
222	Glasser_R_7AL
223	Glasser_R_SCEF
224	Glasser_R_6ma
225	Glasser_R_7Am
226	Glasser_R_7PL
227	Glasser_R_7PC
228	Glasser_R_LIPv
229	Glasser_R_VIP
230	Glasser_R_MIP
231	Glasser_R_1
232	Glasser_R_2
233	Glasser_R_3a
234	Glasser_R_6d
235	Glasser_R_6mp
236	Glasser_R_6v

237	Glasser_R_p24pr
238	Glasser_R_33pr
239	Glasser_R_a24pr
240	Glasser_R_p32pr
241	Glasser_R_a24
242	Glasser_R_d32
243	Glasser_R_8BM
244	Glasser_R_p32
245	Glasser_R_10r
246	Glasser_R_47m
247	Glasser_R_8Av
248	Glasser_R_8Ad
249	Glasser_R_9m
250	Glasser_R_8BL
251	Glasser_R_9p
252	Glasser_R_10d
253	Glasser_R_8C
254	Glasser_R_44
255	Glasser_R_45
256	Glasser_R_47l
257	Glasser_R_a47r
258	Glasser_R_6r
259	Glasser_R_IFJa
260	Glasser_R_IFJp
261	Glasser_R_IFSp
262	Glasser_R_IFSa
263	Glasser_R_p9-46v
264	Glasser_R_46
265	Glasser_R_a9-46v
266	Glasser_R_9-46d
267	Glasser_R_9a
268	Glasser_R_10v
269	Glasser_R_a10p
270	Glasser_R_10pp
271	Glasser_R_11l
272	Glasser_R_13l
273	Glasser_R_OFC
274	Glasser_R_47s
275	Glasser_R_LIPd
276	Glasser_R_6a
277	Glasser_R_i6-8
278	Glasser_R_s6-8
279	Glasser_R_43
280	Glasser_R_OP4
281	Glasser_R_OP1
282	Glasser_R_OP2-3
283	Glasser_R_52
284	Glasser_R_RI

285	Glasser_R_PFcm
286	Glasser_R_PoI2
287	Glasser_R_TA2
288	Glasser_R_FOP4
289	Glasser_R_MI
290	Glasser_R_Pir
291	Glasser_R_AVI
292	Glasser_R_AAIC
293	Glasser_R_FOP1
294	Glasser_R_FOP3
295	Glasser_R_FOP2
296	Glasser_R_PFt
297	Glasser_R_AIP
298	Glasser_R_EC
299	Glasser_R_PreS
300	Glasser_R_H
301	Glasser_R_ProS
302	Glasser_R_PeEc
303	Glasser_R_STGa
304	Glasser_R_PBelt
305	Glasser_R_A5
306	Glasser_R_PHA1
307	Glasser_R_PHA3
308	Glasser_R_STSda
309	Glasser_R_STSdp
310	Glasser_R_STSvp
311	Glasser_R_TGd
312	Glasser_R_TE1a
313	Glasser_R_TE1p
314	Glasser_R_TE2a
315	Glasser_R_TF
316	Glasser_R_TE2p
317	Glasser_R_PHT
318	Glasser_R_PH
319	Glasser_R_TPOJ1
320	Glasser_R_TPOJ2
321	Glasser_R_TPOJ3
322	Glasser_R_DVT
323	Glasser_R_PGp
324	Glasser_R_IP2
325	Glasser_R_IP1
326	Glasser_R_IP0
327	Glasser_R_PFop
328	Glasser_R_PF
329	Glasser_R_PFm
330	Glasser_R_PGi
331	Glasser_R_PGs
332	Glasser_R_V6A

333	Glasser_R_VMV1
334	Glasser_R_VMV3
335	Glasser_R_PHA2
336	Glasser_R_V4t
337	Glasser_R_FST
338	Glasser_R_V3CD
339	Glasser_R_LO3
340	Glasser_R_VMV2
341	Glasser_R_31pd
342	Glasser_R_31a
343	Glasser_R_VVC
344	Glasser_R_25
345	Glasser_R_s32
346	Glasser_R_pOFC
347	Glasser_R_PoI1
348	Glasser_R_Ig
349	Glasser_R_FOP5
350	Glasser_R_p10p
351	Glasser_R_p47r
352	Glasser_R_TGv
353	Glasser_R_MBelt
354	Glasser_R_LBelt
355	Glasser_R_A4
356	Glasser_R_STSva
357	Glasser_R_TE1m
358	Glasser_R_PI
359	Glasser_R_a32pr
360	Glasser_R_p24
504	HO_Left_Thalamus
505	HO_Left_Caudate
506	HO_Left_Putamen
507	HO_Left_Pallidum
509	HO_Left_Hippocampus
510	HO_Left_Amygdala
511	HO_Left_Accumbens
515	HO_Right_Thalamus
516	HO_Right_Caudate
517	HO_Right_Putamen
518	HO_Right_Pallidum
519	HO_Right_Hippocampus
520	HO_Right_Amygdala
521	HO_Right_Accumbens

5. Supplementary references

1. T. N. Tombaugh, Trail Making Test A and B: normative data stratified by age and education. *Archives of clinical neuropsychology* **19**, 203-214 (2004).
2. S. Lehrl, G. Triebig, B. Fischer, Multiple choice vocabulary test MWT as a valid and short test to estimate premorbid intelligence. *Acta Neurologica Scandinavica* **91**, 335-345 (1995).
3. S. R. Kay, A. Fiszbein, L. A. Opler, The positive and negative syndrome scale (PANSS) for schizophrenia. *Schizophrenia bulletin* **13**, 261-276 (1987).
4. A. T. Beck, C. H. Ward, M. Mendelson, J. Mock, J. Erbaugh, An inventory for measuring depression. *Arch Gen Psychiatry* **4**, 561-571 (1961).
5. U. Braun *et al.*, Dynamic reconfiguration of frontal brain networks during executive cognition in humans. *Proc Natl Acad Sci U S A* **112**, 11678-11683 (2015).
6. M. M. Plichta *et al.*, Test-retest reliability of evoked BOLD signals from a cognitive-emotive fMRI test battery. *Neuroimage* **60**, 1746-1758 (2012).
7. J. H. Callicott *et al.*, Physiological characteristics of capacity constraints in working memory as revealed by functional MRI. *Cerebral Cortex* **9**, 20-26 (1999).
8. M. F. Glasser *et al.*, A multi-modal parcellation of human cerebral cortex. *Nature* **536**, 171-178 (2016).
9. S. M. Smith *et al.*, Advances in functional and structural MR image analysis and implementation as FSL. *Neuroimage* **23 Suppl 1**, S208-219 (2004).
10. R. F. Galán, On how network architecture determines the dominant patterns of spontaneous neural activity. *PloS one* **3** (2008).
11. C. Honey *et al.*, Predicting human resting-state functional connectivity from structural connectivity. *Proc Natl Acad Sci U S A* **106**, 2035-2040 (2009).
12. T. M. Karrer *et al.*, A practical guide to methodological considerations in the controllability of structural brain networks. *J Neural Eng* 10.1088/1741-2552/ab6e8b (2020).
13. J. Kim *et al.*, Topological Principles of Control in Dynamical Network Systems. *arXiv preprint arXiv:1702.00354* (2017).
14. J. Z. Kim, D. S. Bassett, Linear dynamics & control of brain networks. *arXiv preprint arXiv:1902.03309* (2019).
15. R. F. Betzel, S. Gu, J. D. Medaglia, F. Pasqualetti, D. S. Bassett, Optimally controlling the human connectome: the role of network topology. *Sci Rep* **6**, 30770 (2016).
16. J. Z. Kim *et al.*, Role of Graph Architecture in Controlling Dynamical Networks with Applications to Neural Systems. *Nat Phys* **14**, 91-98 (2018).
17. M. Loh, E. T. Rolls, G. Deco, A dynamical systems hypothesis of schizophrenia. *PLoS Comput Biol* **3**, e228 (2007).
18. E. T. Rolls, M. Loh, G. Deco, G. Winterer, Computational models of schizophrenia and dopamine modulation in the prefrontal cortex. *Nat Rev Neurosci* **9**, 696-709 (2008).
19. D. Durstewitz, J. K. Seamans, The dual-state theory of prefrontal cortex dopamine function with relevance to catechol-o-methyltransferase genotypes and schizophrenia. *Biol Psychiatry* **64**, 739-749 (2008).
20. L. Fazio *et al.*, Transcriptomic context of DRD1 is associated with prefrontal activity and behavior during working memory. *Proceedings of the National Academy of Sciences of the United States of America* 10.1073/pnas.1717135115 (2018).
21. P. Selvaggi *et al.*, Genetic Variation of a DRD2 Co-expression Network is Associated with Changes in Prefrontal Function After D2 Receptors Stimulation. *Cerebral cortex* 10.1093/cercor/bhy022 (2018).
22. G. Pergola *et al.*, Prefrontal co-expression of schizophrenia risk genes is associated with treatment response in patients. *bioRxiv* (2018).
23. G. Pergola *et al.*, DRD2 co-expression network and a related polygenic index predict imaging, behavioral and clinical phenotypes linked to schizophrenia. *Transl Psychiatry* **7**, e1006 (2017).
24. B. Zhang, S. Horvath, A general framework for weighted gene co-expression network analysis. *Statistical applications in genetics and molecular biology* **4**, Article17 (2005).

25. C. Colantuoni *et al.*, Temporal dynamics and genetic control of transcription in the human prefrontal cortex. *Nature* **478**, 519-523 (2011).
26. G. Pergola *et al.*, Combined effect of genetic variants in the GluN2B coding gene (GRIN2B) on prefrontal function during working memory performance. *Psychological medicine* **46**, 1135-1150 (2016).
27. L. Fazio *et al.*, Transcriptomic context of DRD1 is associated with prefrontal activity and behavior during working memory. *Proc Natl Acad Sci U S A* **115**, 5582-5587 (2018).
28. P. Selvaggi *et al.*, Genetic Variation of a DRD2 Co-expression Network is Associated with Changes in Prefrontal Function After D2 Receptors Stimulation. *Cereb Cortex* **29**, 1162-1173 (2019).
29. E. M. Gordon *et al.*, Generation and Evaluation of a Cortical Area Parcellation from Resting-State Correlations. *Cereb Cortex* **26**, 288-303 (2016).
