# Supplementary material for: Brain network dynamics during working memory are modulated by dopamine and diminished in schizophrenia
Source: Nat Commun. 2021 Jun 9;12:3478. doi: 10.1038/s41467-021-23694-9 (PMC8190281; doi:10.1038/s41467-021-23694-9)
Supplement: Supplementary file 3 — Reporting Summary [file 41467_2021_23694_MOESM3_ESM.pdf]

nature research

Corresponding author(s): Urs Braun

Last updated by author(s): Nov 20, 2020

Reporting Summary

Nature Research wishes to improve the reproducibility of the work that we publish. This form provides structure for consistency and transparency in reporting. For further information on Nature Research policies, see Authors & Referees and the Editorial Policy Checklist.

Statistics

For all statistical analyses, confirm that the following items are present in the figure legend, table legend, main text, or Methods section.

n/a	Confirmed	
☐	☒	The exact sample size (n) for each experimental group/condition, given as a discrete number and unit of measurement
☐	☒	A statement on whether measurements were taken from distinct samples or whether the same sample was measured repeatedly
☐	☒	The statistical test(s) used AND whether they are one- or two-sided<br>*Only common tests should be described solely by name; describe more complex techniques in the Methods section.*
☐	☒	A description of all covariates tested
☐	☒	A description of any assumptions or corrections, such as tests of normality and adjustment for multiple comparisons
☐	☒	A full description of the statistical parameters including central tendency (e.g. means) or other basic estimates (e.g. regression coefficient) AND variation (e.g. standard deviation) or associated estimates of uncertainty (e.g. confidence intervals)
☐	☒	For null hypothesis testing, the test statistic (e.g. F, t, r) with confidence intervals, effect sizes, degrees of freedom and P value noted<br>*Give P values as exact values whenever suitable.*
☒	☐	For Bayesian analysis, information on the choice of priors and Markov chain Monte Carlo settings
☒	☐	For hierarchical and complex designs, identification of the appropriate level for tests and full reporting of outcomes
☐	☒	Estimates of effect sizes (e.g. Cohen's d, Pearson's r), indicating how they were calculated

Our web collection on statistics for biologists contains articles on many of the points above.

Software and code

Policy information about availability of computer code

Data collection	Siemens Trio Software version Syngo VB15-17 (Siemens Erlangen, Germany), Presentation versions 11.0 to 16.0 (https://www.neurobs.com)
Data analysis	SPSS (IBM), version 24.0.0.0; R (https://www.Rproject.org/); MATLAB and Statistics Toolbox Release 2013b, The MathWorks, Inc., Natick, Massachusetts, United States., SPM12 (https://www.fil.ion.ucl.ac.uk/spm/), FSL Version 5.0.10 (https://fsl.fmrib.ox.ac.uk/fsl/fslwiki), DSI studio february 3 2017 build (http://dsi-studio.labsolver.org), custom code (https://www.dropbox.com/sh/kvd4oxujefkt3mr/AADKg71wPHS2PsNhmv-PBAOZa?dl=0)

For manuscripts utilizing custom algorithms or software that are central to the research but not yet described in published literature, software must be made available to editors/reviewers. We strongly encourage code deposition in a community repository (e.g. GitHub). See the Nature Research guidelines for submitting code & software for further information.

Data

Policy information about availability of data

All manuscripts must include a data availability statement. This statement should provide the following information, where applicable:

- Accession codes, unique identifiers, or web links for publicly available datasets
- A list of figures that have associated raw data
- A description of any restrictions on data availability

Data supporting the findings of this study are available upon reasonable request from the corresponding author. Data sharing underlies GDPR restrictions. The figures 2-3 and S2-4 have associated raw data.

Field-specific reporting

Please select the one below that is the best fit for your research. If you are not sure, read the appropriate sections before making your selection.

☐ Life sciences ☒ Behavioural & social sciences ☐ Ecological, evolutionary & environmental sciences

For a reference copy of the document with all sections, see nature.com/documents/nr-reporting-summary-flat.pdf

Behavioural & social sciences study design

All studies must disclose on these points even when the disclosure is negative.

Study description	Quantitative study combining functional magnetic resonance imaging, genetic analyses and pharmacological interventions.
Research sample	We aimed to investigate neural mechanisms of working memory function in healthy, unaffected individuals of the general population and thus recruited a a representative, community-based sample of healthy individuals (n = 178, age: 33.05 +/- 10.98, 85 females). In addition, we aimed to investigate the same mechanisms in schizophrenia patients and therefore recruited a sample of schizophrenia patients (n = 24, age: 32.25 +/- 10.33, 6 females). In the pharmacological study, we aimed to investigate the modulation of brain functions through directed challenge of the dopaminergic system in healthy volunteers and therefore recruited a sample of young, mentally and somatic healthy individuals (n = 16, age: 26.63 +/- 5.34, 8 females).
Sampling strategy	Healthy participants were recruited via newspaper, website and board advertisements, using a combination of voluntary response sampling and stratification sampling. The patients were recruited from the Department of Psychiatry and Psychotherapy at the Central Institute of Mental Health in Mannheim and via local advertisements. A trained psychiatrist or psychologist verified the diagnosis of schizophrenia based on ICD-10 criteria. Sample sizes were determined based on previous fMRI studies and pharmacological interventions trials. For the genetic association study, please see a detailed discussion on sample size estimation in the supplemental material.
Data collection	Blood-oxygen-level-dependent fMRI was performed on a 3 Tesla Siemens Trio scanner using an echo-planar-imaging sequence. Blood was acquired on the same day as scanning took place. Scanning was executed by a researcher, a research assistant and medical technical assistant, who where blinded to the research goals and - in case of the pharmacological study - experimental condition. Psychological assessment was done by pen-and-pencil questionnaires and computerized tests were administered to assess neuropsychological function by researchers and research assistants. Further details on all methods are provided in the ONLINE METHODS.
Timing	Participants were recruited from September 2010 to January 2017 at the Central Institute of Mental Health (CIMH) in Mannheim, Germany.
Data exclusions	One subject was excluded from the analysis due to an excessive body-mass index (BMI > 30) as indicated in the pre-established exclusion criteria. One participant did not complete all three sessions of the pharmacological study.
Non-participation	One participant did not complete all three sessions of the pharmacological study.
Randomization	The order of the pharmacological interventions was randomized.

Reporting for specific materials, systems and methods

We require information from authors about some types of materials, experimental systems and methods used in many studies. Here, indicate whether each material, system or method listed is relevant to your study. If you are not sure if a list item applies to your research, read the appropriate section before selecting a response.

Materials & experimental systems

n/a	Involved in the study	
☒	☐	Antibodies
☒	☐	Eukaryotic cell lines
☒	☐	Palaeontology
☒	☐	Animals and other organisms
☐	☒	Human research participants
☒	☐	Clinical data

Methods

n/a	Involved in the study	
☒	☐	ChIP-seq
☒	☐	Flow cytometry
☐	☒	MRI-based neuroimaging

Human research participants

Policy information about studies involving human research participants

Population characteristics	see above "Behavioural & social sciences study design" and Table 1 and 2 for detailed participant characteristics
Recruitment	We recruited a a representative, community-based sample of healthy individuals via newspaper, website and board

Recruitment

advertisements. Interested individuals were screened for inclusion and exclusion criteria via telephone. The patients were recruited from the Department of Psychiatry and Psychotherapy at the Central Institute of Mental Health in Mannheim and via local advertisements and screened for inclusion and exclusion criteria on the ward or via telephone. Both, healthy controls and patients received expense allowance. To the best of our knowledge, there was no self-selection bias.

Ethics oversight

Medical Ethics Committee II of the Medical Faculty Mannheim at the Ruprecht-Karls-University in Heidelberg, Germany

Note that full information on the approval of the study protocol must also be provided in the manuscript.

Magnetic resonance imaging

Experimental design

Design type

N-back: a well-established working memory paradigm consisting of a high memory load (2-back) and an attention control condition requiring motor response (0-back), block design.

Design specifications

Block design: a diamond-shaped stimulus containing a number from 1 to 4 was presented every 2 seconds. In the 0-back condition, subjects were required to press the button on the response box corresponding to the number currently displayed on the presentation screen. In the 2-back condition, subjects were required to press the button on the response box corresponding to the number presented two stimuli before the number currently displayed on the presentation screen. Stimuli were presented in alternating blocks of either 0-back or 2-back conditions. In each condition block, 14 stimuli were presented. Each condition block was repeated 4 times.

Behavioral performance measures

Task performance was measured by accuracy (defined as the percent of correct answers) and reaction time (defined as the time span between stimulus onset and button press) for each condition separately

Acquisition

Imaging type(s)

functional

Field strength

3

Sequence & imaging parameters

study 1: TR/ TE = 2000/30 ms, α =80°, 28 axial slices (slice-thickness = 4 mm + 1 mm gap), descending acquisition, FoV = 192 mm, acquisition matrix = 64 x 64 128 volumes
study 2 (pharmacological study): TR = 1790 ms, TE = 28 ms, 34 axial slices per volume, voxel size = 3 x 3 x 3 mm, 1 mm gap, 192 x 192 mm field of view, 76° flip angle, descending acquisition.

Area of acquisition

whole brain

Diffusion MRI ☒ Used ☐ Not used

Parameters

TR 14000ms, TE 86ms, 2mm slice thickness, 60 non-collinear directions, b-value 1000 s/mm2, 1 b0 image, FOV 256 mm.

Preprocessing

Preprocessing software

SPM 12 and DTI studio

Normalization

direct

Normalization template

Montreal Neurological Institute (MNI) space with resampling to 3 × 3 × 3 mm voxels

Noise and artifact removal

6 rigid-body-transform motion parameters and time-courses from white-matter und cerebro-spinal fluid were used as nuissance regressors

Volume censoring

We used standard procedures for motion correction in activation analysis as implemented in SPM 12.

Statistical modeling & inference

Model type and settings

Standard first level GLM models included regressors for the 0-back and 2-back conditions of interest, as well as the 6 motion parameters as regressors of non-interest.

Effect(s) tested

To define brain activity pattern associated with each condition of the task, we extracted GLM (beta) parameter estimates for the 0-back and 2-back conditions separately and we averaged them across all voxels in each of the 374 regions.

Specify type of analysis: ☒ Whole brain ☐ ROI-based ☐ Both

Statistic type for inference
(See Eklund et al. 2016)

For the main analysis, no conventional mass univariate analyses were conducted, instead we used standard t-tests and regression analysis on the output parameters from the network control analysis.
In the supplement, we provide the results of more conventional mass-univariate approaches reporting peak voxel results after family-wise error correction in a predefined ROI.

Correction

For the supplemental analyses using conventional mass-univariate approaches, we used family-wise error correction.

Models & analysis

n/a	Involved in the study
☒	☐ Functional and/or effective connectivity
☒	☐ Graph analysis
☒	☐ Multivariate modeling or predictive analysis
